# Supplementary material for: Data-dependent visualization of biological networks in the web-browser with NDExEdit
Source: PLoS Comput Biol. 2022 Jun 8;18(6):e1010205. doi: 10.1371/journal.pcbi.1010205 (PMC9212158; doi:10.1371/journal.pcbi.1010205)
Supplement: Attachment — Submitted filename: Response to Reviewers.pdf [file pcbi.1010205.s001.pdf]

Response to Reviewers

Dear Reviewers,

thank you for taking the time to evaluate our manuscript and giving us the opportunity to submit a revised version. We appreciate the effort that you dedicated to providing us with thorough feedback and are grateful for the insightful comments on our manuscript. Your critical but extraordinary helpful responses are extremely valuable to improve our paper.

The manuscript was updated to adhere to the standard Software article layout and order of the Abstract, Introduction, Author Summary, Design and Implementation, Results, Availability and Future Directions. Because of this we included an extended the conclusive part into the results section to point out the advantages of NDExEdit more clearly and in more detail.

An update to our application resolved a major concern: NDExEdit now allows the full workflow of opening a network from NDEx, editing it, and saving the results back on the platform. Our application has now the option to browse public and private networks on NDEx, as well as to create a new or update an existing network. We also adapted those changes in our manuscript at the appropriate passages and updated the “limitations” section. Again, thank you for not only illustrating this shortcoming, its implication on the whole workflow, and subsequently on the significance of our application but also pointing us in the right direction to resolve this issue!

By resolving the above shortcoming we could also improve the significance and novelty within the manuscript. This also helped to illustrate the differences to Cytoscape which are now included as a subsection in the manuscript. We modified the existing section to not only discuss the limitations of NDExEdit but also those of Cytoscape which can be surmounted by NDExEdit. We put special focus on how it can be useful in a collaborative workflow involving multiple users. In this multi-user environment, the single users with their private instance of NDExEdit in the web browser can log in to the NDEx platform, which indirectly provides the user management.

All minor concerns have been resolved: The failing of the validation while uploading the example network to NDEx, as well as the failure of discrete mappings, was due to a bug in the CX creation that we resolved shortly after discovery. This may also have caused the unexplained locking and disabling while editing mappings because the data structure was disrupted. The 404 error after refreshing was probably caused by an accompanying bug which is also resolved.

We also tried to put more emphasis on the point that NDExEdit is a web application that builds on top of cytoscape.js, rather than being a library competing with it or any other. The decision for cytscape.js, therefore, was based on the relations to, and between Cytoscape and NDEx to provide a consistent visualization of networks. Accordingly, the color scheme for mappings is also adopted consistently by using the standard browser-based gradients.

The limitations of NDExEdit in its scalability are not easy to be estimated: Since it is a browser-based application it is mainly limited by the memory restrictions of the used web browser. However, more limiting is its intended use: At a certain size of a network, a browser-based visualization would simply not be adequate anymore. As a practical solution, NDExEdit uses a boundary by the number of nodes and edges within a network similar to NDEx.
